# Prevalence of symptomatic hip, knee, and spine osteoarthritis nationwide health survey analysis of an elderly Korean population

**DOI:** 10.1097/MD.0000000000006372

**Published:** 2017-03-24

**Authors:** Jung-Ho Park, Jae-Young Hong, Kyungdo Han, Seung-Woo Suh, Si-Young Park, Jae-Hyuk Yang, Seung-Woo Han

**Affiliations:** a Department of Orthopedics, Korea University Ansan Hospital, Ansan; b Department of Biostatistics, College of Medicine, Catholic University; c Department of Orthopedics, Korea University Guro Hospital; d Department of Orthopedics, Korea University Anam Hospital, Seoul, South Korea.

**Keywords:** hip, knee, osteoarthritis, prevalence, related factors, spine

## Abstract

Osteoarthritis is prominent among the elderly, with symptoms originating from multiple parts of the body. A cross-sectional study of a nationwide survey was performed to describe the prevalence of and identify factors related to symptomatic hip, knee, and spine osteoarthritis.

This cross-sectional study collected data from the Fifth Korean National Health and Nutrition Examination Survey (KNHANES V-5; 2010–2012). After excluding ineligible subjects, there were 8976 subjects in this study (3830 males and 5146 females). All subjects reported symptoms and disabilities related to osteoarthritis. Plain radiographs of the spine, hip, and knee were taken in all subjects.

Overall, 9.3% of male participants and 28.5% of female participants were diagnosed with symptomatic osteoarthritis according to survey criteria. Women showed a significantly higher prevalence in all age groups (*P* < 0.05). Multiple-joint osteoarthritis was diagnosed in 10.8% of male patients and 22.8% of female patients with osteoarthritis. Several demographic and lifestyle variables were related to osteoarthritis morbidity. Anthropometric and laboratory measurements were also related to osteoarthritis morbidity. In addition, mental distress and quality of life were significantly compromised in osteoarthritis. There were more significant relationships for these factors among women with a higher prevalence of multijoint osteoarthritis.

A significant proportion of the elderly with single- or multiple-joint osteoarthritis had a variety of pain origins that were closely related. Osteoarthritis was also significantly related to several factors, including mental distress and quality of life.

## 1 Introduction

Advancements in the diagnosis and treatment of disease have led to an increase in the mean age of the population. More people now experience degenerative osteoarthritis (OA), which can occur in several mobile joints of the human body including the hip, knee, and spine. However, symptoms associated with OA may be similar to those due to other origins.^[1–7]^ To diagnose and treat degenerative OA, physicians must be familiar with the characteristics and prevalence of OA as well as factors related to developing OA.^[5,8–18]^ In this study, we analyzed a large cross-sectional population to determine the prevalence and characteristics of the most common forms of degenerative arthritis (e.g., of the hip, knee, and spine). The aim of this study was to investigate the prevalence and related factors of multijoint OA using data from the Korean National Health and Nutrition Examination Survey (KNHANES).

## 2 Materials and methods

### 2.1 Study population

**Figure 1:**
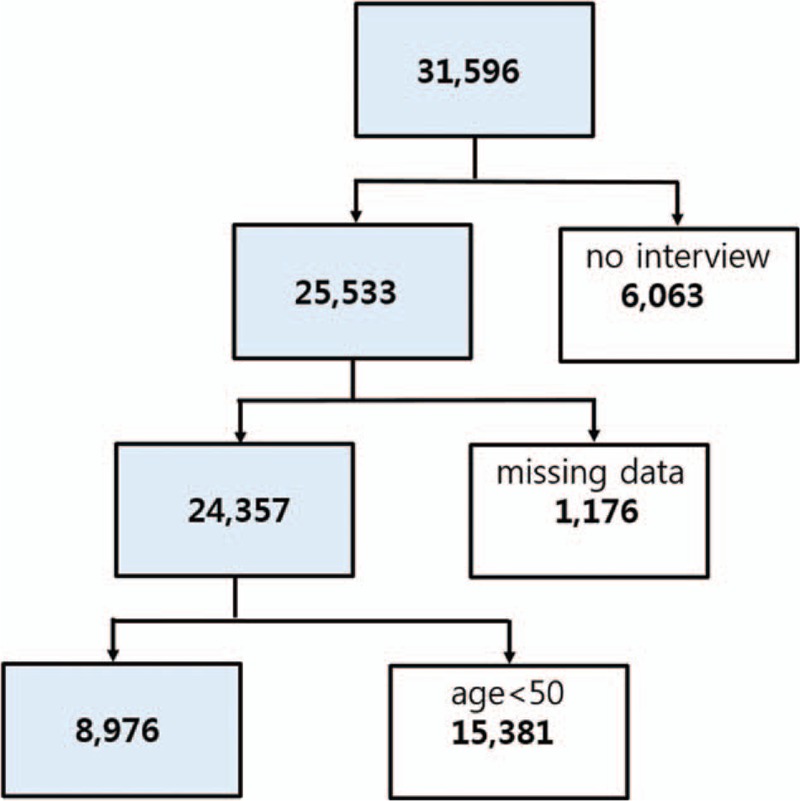
Flowchart showing inclusion and exclusion of subjects according to study criteria.

The study design was cross-sectional and involved 3 years of data from the Fifth Korean National Health and Nutrition Examination Survey (KNHANES-V: 2010–2012). The KNHANES is a nationwide health and nutrition survey of children, adolescents, and adults that has been regularly conducted since 1998 by the Korean Centers for Disease Control and Prevention.^[19]^ The survey was performed by the Korean Ministry of Health and Welfare, and was designed to assess national health and nutritional levels. A stratified, multistage probability sampling design was used, and sampling units were based on geographical area, age, and sex. Among a total of 31,596 subjects (Fig. 1), 25,533 (80.8%) completed the health interview survey and health examination survey. Initially, subjects who underwent radiographic examinations without a health interview were excluded (n = 6063). Persons younger than 50 years (n = 15,381) and those with missing data for variables included in the analysis (n = 1176) were excluded. The remaining 8976 subjects who met the inclusion criteria (3830 males and 5146 females) had undergone physical and laboratory examinations, including radiographic examination of the knee, hip, and spine. Health interview data were retrieved from the KNHANES, including demographic and lifestyle variables (physical activity and mental status). All subjects provided written informed consent, and the Korea Centers for Disease Control and Prevention Institutional Review Board (ethical review committee for health survey data) approved the study protocol (IRB No 2010-02CON-21-C, 2011-02CON-06-C, 2012-01EXP-01-2C).

### 2.2 Radiographic examination and symptom assessment

Radiographic examinations of the knee, hip, and spine were taken using a SD3000 Synchro Stand (Accele Ray, Switzerland). Bilateral anteroposterior, weight-bearing anteroposterior, and lateral (30° of flexion) plain radiographs of the knees were obtained. Bilateral anteroposterior and lateral pain radiographs of the hips were obtained. Anteroposterior and lateral plain radiographs of the spine were obtained. Radiographic changes in each joint were then independently assessed by 2 radiologists using the Kellgren/Lawrence (KL) grading system: Grade 0, none: no visible features of OA; Grade 1, doubtful: questionable osteophytes or questionable joint space narrowing; Grade 2, minimal: definitive small osteophytes, minimal/mild joint space narrowing; Grade 3, moderate: definitive moderate osteophytes, joint space narrowing of at least 50%; Grade 4, severe: severely impaired joint space, subchondral bone cysts and sclerosis.^[20]^ The presence of radiographic OA was defined as KL grade≥2. If grades given by the 2 radiologists were discrepant by 1 KL grade for the same case, the higher grade was accepted. If grades were discrepant by more than 2 grades, a third radiologist was consulted, and the grade assigned by the third radiologist was accepted. The concordance rate within 1 KL grade of knee joints for the same case was 94.76%. The weighted kappa coefficient for knee x-rays was 0.65, which demonstrated very high inter-rater agreement. All subjects described their current symptoms related to each joint (e.g., hip, knee, and spine), and symptoms were scored. Subjects who had experienced arthritic pain of each joint for more than 30 days in the past 3 months were asked to report the average intensity of pain, regardless of pain medication, using an 11-point numeric rating scale (NRS) ranging from 0 to 10, with higher scores indicating a higher level of intensity.

### 2.3 Demographic and lifestyle variables

Demographic variables included age (years), sex, equivalized monthly household income, marital status, current residence, education level, smoking status (never smoked, past smoker, or current smoker), alcohol consumption (grams alcohol/d), and physical activity (low, moderate, or high). Equivalized household income was calculated as the total monthly household income divided by the square root of the total number of household members. Average alcoholic beverage consumption was assessed by self-reported questionnaire, and then converted to the amount of pure alcohol consumed per day. Subjects were classified into 3 groups according to the amount of alcohol consumption per day (g/day) during the 1-month period before the interview: nondrinkers, light drinkers (<15 g/day), and moderate-to-heavy drinkers (≥15 g/day). Subjects who had smoked more than 100 cigarettes in their lifetime were classified as ever-smokers. Physical activity was quantified as the metabolic equivalent of task minutes per week (MET-minutes per week), which was calculated using the scoring protocol of the Korean version of the International Physical Activity Questionnaire short form.^[21]^ Accordingly, physical activity levels were classified as low (<600 MET-minutes per week), moderate (600 to 3000 MET-minutes per week), or high (>3000 MET-minutes per week).

### 2.4 Anthropometric and laboratory measurements

Body weight and height were measured, and the body mass index was calculated using the following formula: body weight (kg) / height^2^ (m^2^). Body weight and height were measured using standard protocols. Waist circumference was measured at the narrowest point, between the lower costal margin and the iliac crest, during exhalation. General obesity was defined as body mass index ≥ 25, and the cutoff points for central obesity were defined as WC ≥ 90 cm for men and WC ≥ 85 cm for women.^[22]^ Systolic blood pressure and diastolic blood pressure were measured 3 separate times at 5-min intervals using a standard mercury sphygmomanometer. After each participant's BP had been manually measured 3 times by well-trained observers using a mercury sphygmomanometer (Baumanometer; Baum, Copiague, NY), the average values of the second and third measurements were used in the analysis. Hypertension was defined as blood pressure ≥ 140/90 mm Hg or current use of antihypertensive medication. Blood samples were obtained from the antecubital vein of each participant after at least 8 hours of fasting, and random midstream urine samples were collected from subjects. Samples were processed and immediately refrigerated, then transported in cold storage units to the Central Testing Institute in Seoul, Korea, and analyzed within 24 hours of transportation. Fasting plasma glucose, total cholesterol, and triglyceride levels were measured by enzymatic methods using a Hitachi Automatic Analyzer 7600 (Hitachi, Tokyo, Japan). Serum and urine creatinine levels were measured by kinetic colorimetry using a Hitachi Automatic Analyzer 7600. The estimated glomerular filtration rate (GFR) was calculated using the equation from the Modification of Diet in Renal Disease study.^[23]^ Subjects were considered to have diabetes if they had a fasting blood glucose of ≥126 mg/dL or if they were taking antidiabetic medication.

### 2.5 Mental status and quality of life variables

To assess the mental health of the study population, surveys provided to the subjects included the same questions as those in the KNHANES surveys.^[24]^ Three dimensions within the domains of health status and mental health were measured: stress, depression, and suicidal thoughts and attempts. Subjects reported their level of stress as none, mild, moderate, or severe. Depression was screened using the Korean version of the World Health Organization Composite International Diagnostic Interview-Short Form, which was validated as a cost-effective screening instrument that is easily integrated into health surveys.^[24]^ To assess depression, participants answered “yes” or “no” to one of the following questions about whether they had experienced a depressed mood for 2 or more continuous weeks during the previous year: “In your lifetime, have you ever had 2 weeks or more when nearly every day you felt sad, blue, or depressed?” and “Have there ever been 2 weeks or longer when you lost interest in most things such as work or hobbies or things you usually like to do for fun?” Suicidal ideation was assessed by the question “In the last 12 months, did you think about committing suicide?” A “yes” or “no” response was used to determine whether the subject had suicidal thoughts. Health-related quality of life (HRQOL) was evaluated based on the EQ-5D, a self-reported questionnaire widely used to assess the quality of life of a general population. The Korean EQ-5D was developed according to guidelines from the EQ group. Its reliability and validity have been tested in patients with rheumatism, cancer, and chronic disease.^[25,26]^ The EQ-5D consists of 5 questions evaluating a respondent's current health status in terms of mobility, self-care, usual activities, pain/discomfort, and anxiety/depression. In each dimension, a respondent can belong to 1 of 3 categories, and these are classified as “no problems” and “problems,” including moderate to severe problems.^[8,23,27]^

### 2.6 Statistical analysis

Statistical analyses were conducted using SAS survey procedures (version 9.3; SAS Institute, Cary, NC) in a manner that reflected sampling weights and provided nationally representative estimates. The characteristics of subjects with OA were compared with those of subjects without OA (control group) using 2 independent sample *t*-tests and 1-way analysis of variance for continuous variables and the chi-squared test for categorical variables. Multivariate logistic regression analyses were conducted to investigate the relationship between parameters. SES variables with *P*-values <0.15 in univariate analyses were selected for multivariate analyses. A *P*-value <0.05 was considered statistically significant.

## 3 Results

### 3.1 Symptoms and radiographic findings

OA was defined as “OA in at least 1 joint on plain radiographs with related pain including hip, knee, or spine.” Radiographic changes of each joint were assessed, and the presence of radiographic OA was defined as KL grade ≥2. Subjects who had experienced arthritic pain of each joint for more than 30 days in the past 3 months were included in the OA group. We used the ACR criteria for knee and hip OA with our criteria to confirm the diagnosis. In cross-match analysis, there were significant mismatches of subjective pain and radiographic findings (Table 1). Thus, the prevalence of OA in males was 0.1%, 4.5%, and 5.6% in the hip, knee, and spine in the study population, respectively. Female groups showed respective prevalence of 0.2%, 19%, and 16% in the hip, knee, and spine, which were significantly greater than in males (chi-squared test, *P* < 0.05).

**Table 1:**
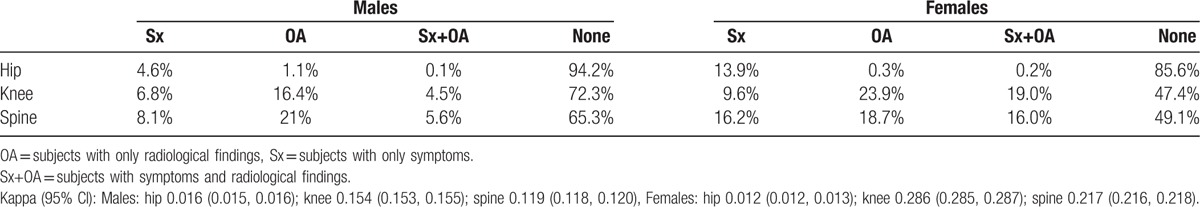
Cross-match analysis of symptoms and radiographic findings.

### 3.2 Prevalence of multijoint OA

Overall, 9.3% of male and 28.5% of female participants were diagnosed with OA (in at least 1 joint) according to the survey (Table 2). The female group showed a significantly higher prevalence in all age groups (chi-squared test, *P* < 0.05). Prevalence progressively increased with age and was highest in the older than 80 years group (male: 33.5%, female: 63.3%). Multijoint OA was defined as “OA in more than 2 joints with related pain including hip, knee, and spine.” Overall, 89.2% of male and 77.2% of female OA patients were diagnosed with single-joint OA, whereas 10.8% of male and 22.8% of female patients were diagnosed with multiple-joint disease (Table 3).

**Table 2:**
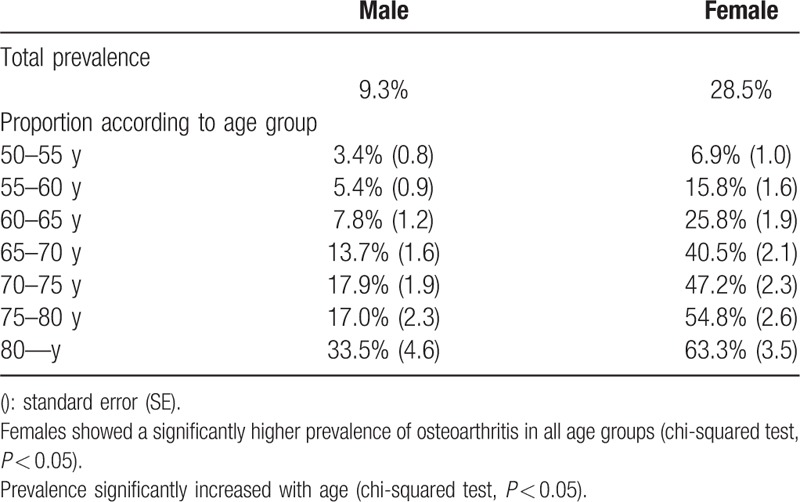
Prevalence of symptomatic osteoarthritis according to age.

**Table 3:**
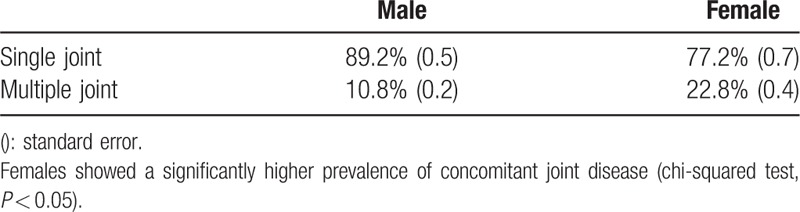
Prevalence of multijoint osteoarthritis including hip, knee, and spine.

### 3.3 Factors related to OA

#### 3.3.1 Demographic and lifestyle variables

Age, living situation, marital status, educational status, and income status were significantly related to OA morbidity (*P* < 0.05; Table 4). Low education and low income were significantly related to OA morbidity (*P* < 0.05). In addition, rural residence and no spouse were significantly related to OA morbidity, especially in females (*P* < 0.05). More significant relationships with these factors were found in females with a higher prevalence of multijoint OA. Smoking, drinking status, and activity level were not significantly related to OA morbidity.

**Table 4:**
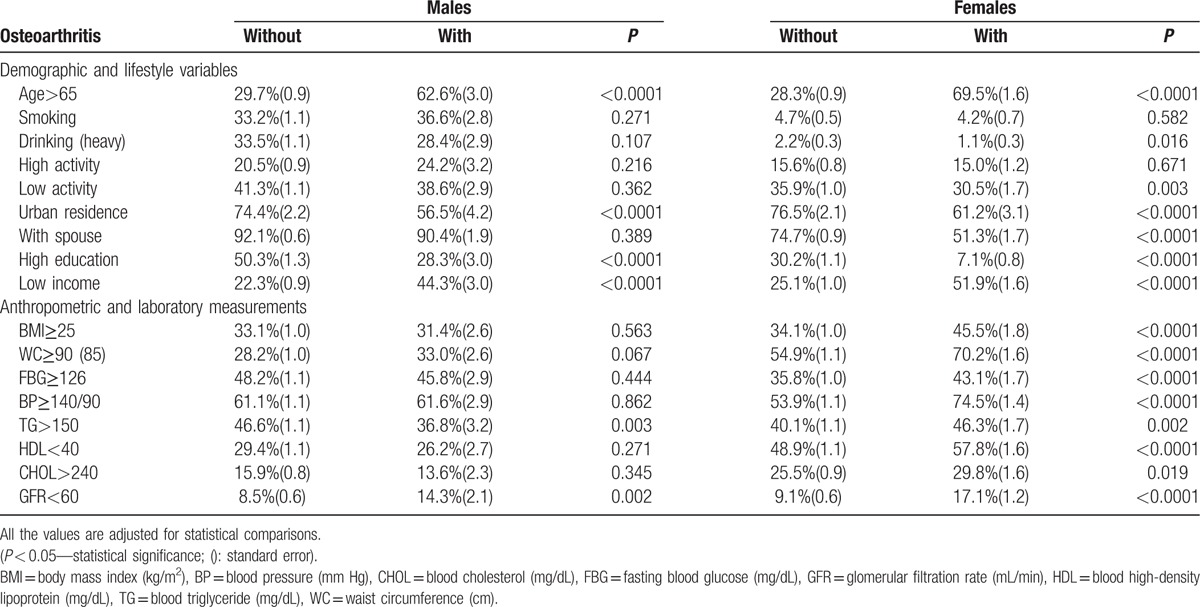
Summary of related factors with symptomatic osteoarthritis according to incidence in 2 groups.

#### 3.3.2 Anthropometric and laboratory measurements

Several anthropometric and laboratory measurements were significantly related to OA, especially in the female and multijoint OA group (*P* < 0.05; Table 4). Body mass index (BMI), waist circumference (WC), fasting blood glucose (FBG), blood pressure (BP), glomerular filtration rate (GFR) and blood levels of triglycerides (TG), high-density lipoprotein (HDL), and cholesterol (CHOL) were significantly related to OA (*P* < 0.05). Significance levels were higher with these factors in females with a higher prevalence of multijoint OA.

#### 3.3.3 Mental status and quality of life variables

There was a significantly higher rate of mental stress in the OA group (Table 5). Mental stress, melancholy, and suicidal thinking were more prevalent in the OA group (*P* < 0.05). In addition, EQL was significantly compromised in the OA group. LQ 1, 2, 3, 4, and 5 were significantly higher in the OA group. Significance levels were higher with these factors in females with a higher prevalence of multijoint OA.

**Table 5:**
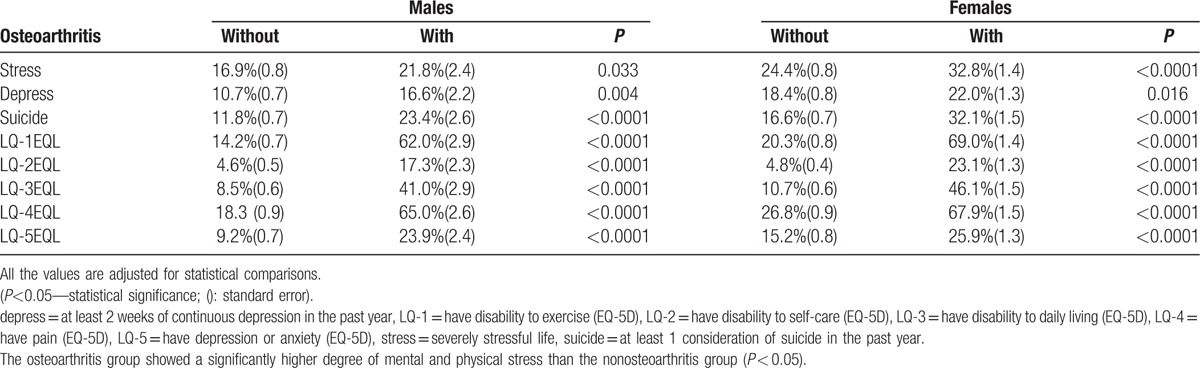
Analysis of symptomatic osteoarthritis and mental status, quality of life.

## 4 Discussion

Because of improved treatment for chronic diseases and lower mortality from infectious diseases, the worldwide population is aging. Older people are now living longer with common and disabling conditions such as OA.^[2–4,7,8,14,28]^ The projected number of older adults with arthritis or other chronic musculoskeletal joint symptoms is expected to nearly double from 21.4 million in 2005 to 41.1 million by 2030 in the United States.^[28]^ In our study, 9.3% of male and 28.5% of female participants were diagnosed with symptomatic degenerative joint OA. There was a higher rate of multijoint OA in the older segment of the population, including hip, knee, and spine joints, which may be comparable to the prevalence of diabetes or hypertension in the elderly. In this study, the prevalence of symptomatic knee OA in males was 4.5%. The female group showed a prevalence of 19% in the knee, which was significantly greater than in males. Muraki et al analyzed 2126 Japanese men and women using a large-scale population-based cohort study. Although they showed a significant difference in the proportion of men and women compared to our study, they reported that the prevalence of symptomatic knee OA was 5.0% in men and 11.3% in women, which is similar to our result.^[6]^ In a European study, Turkiewicz et al^[9]^ reported that the prevalence of frequent knee pain was 25.1%, which was higher in women and similar across age groups. However, they reported that the prevalence of radiographic knee OA was 25.4%, whereas 15.4% had either symptomatic or clinically defined knee OA in a random sample of 10,000 elderly residents of Sweden. Although the incidence in Asian and European studies was similar to that of our study, if we had focused on knee and spine OA, the incidence of multijoint OA may have been even higher.^[11,14,28,31,32]^ Furthermore, we found patients who had radiographic arthritis without symptoms, and vice versa. This selection criterion may have significantly decreased the prevalence, which would have been much higher if we had diagnosed the disease differently.^[6,29,30,33,34]^

There are several studies on the prevalence and incidence of single-joint (e.g., knee) arthritis.^[6,9,11,16,31,37]^ Degenerative OA may occur in several mobile joints of the human body, including the hip, knee, and spine.^[35]^ Symptoms associated with degenerative OA may be similar to those from different origins or different manifestations with the same origin. Thus, a more comprehensive investigation in this field is needed. We analyzed the prevalence and related factors associated with multiple-joint OA. There appears to be a close mechanical relationship between the knee, hip, and spine. Each joint may support body weight and compensate for abnormal motion. In this study, a significant proportion of the population was diagnosed with single- or multijoint OA, including 10.8% of male patients and 22.8% of female patients. However, the prevalence of OA may be underestimated due to the rare incidence of hip joint OA. Problems in 1 joint may affect other joints and induce multijoint OA that should be treated simultaneously. In addition, physicians can misdiagnose the origin of symptoms due to the high prevalence of multijoint OA with similar symptoms. In this study, prevalence progressively increased with age and was the highest in patients more than 80 years old (female: 63.3%). In addition, female patients had a higher prevalence of multijoint OA than males, and several related factors were more closely associated in female patients. It appears that women are more susceptible to multijoint OA than men for several reasons, ^[36–38]^ including hormonal changes and osteoporosis, which may accelerate degenerative changes in multiple joints.

In this study, age, living situation, marital status, educational status, and income status were significantly related to OA morbidity, as were BMI and body composition factors. Previous studies have reported that joint pain is associated with several socio-demographic factors such as sex, old age, low education level, smoking, and occupation.^[39,40]^ In particular, we found more significant relationships with these factors and a higher prevalence of OA in the female group. In this study, living in a rural setting was related to OA. It is possible that participants who live in rural areas may engage in harder labor (e.g., agriculture), which may increase disease risk. The low education level was also associated with OA. Body composition factors had significant relationships to OA, with female dominance. As anticipated, the OA group had a higher BMI with higher blood sugar and lipid levels, which may be the cause or result of generalized OA. Importantly, participants with multijoint OA experienced compromised mental status and psychological dissociation significantly more often. This suggests that in addition to physical pain, mental distress should be treated in this population. Patients in the OA group suffered from significant mental distress and were prone to suicidal thoughts. In terms of life quality and mental health, there were significantly higher rates of mental stress in the disease group. In addition, EQL was significantly decreased in the OA group. In the EQ-5D survey, problems in the domains of daily living activities (e.g., usual activity, mobility, and self-care) and pain/discomfort were significantly more prevalent in persons with OA. Considering that quality of life and mental status are significantly affected by OA, understanding the prevalence and etiology may be important in disease treatment.^[1,6,13,16]^

There are several limitations to this study. First, the cross-sectional study design prevented determination of cause-and-effect relationships. Therefore, future prospective studies are required to better elucidate causal relationships. Second, use of a single 11-point NRS did not allow evaluation of the intensity of acute and chronic pain, including functional impairment. In addition, more sophisticated diagnostic tools (such as MRI and CT) may be needed to evaluate the precise status of joints. Third, the results cannot be confirmed or generalized because of ethnic differences. The prevalence or etiology of OA may be influenced by ethnic or environmental factors, which may decrease the significance of our study. Despite these limitations, this study included a large cross-sectional population with sophisticated statistical methods. A higher number of patients with multiple-joint OA were identified than expected. Our results will be helpful for physicians treating OA.

In conclusion, a significant proportion of patients with OA in a large cross-sectional population had a variety of pain origins that were closely related to each other. OA was significantly related to several factors, including mental distress and quality of life. To prevent misdiagnosis and malpractice, physicians should be aware of the relationships and factors involved in multiple-joint disease, particularly in an elderly population.

## Acknowledgments

The authors used data from the Fifth Korean National Health and Nutrition Examination Survey (KNHANES-V: 2010–2012). The KNHANES is a nationwide health and nutrition survey of children, adolescents, and adults that has been regularly conducted since 1998 by the Korean Centers for Disease Control and Prevention. They acknowledge the Korean Centers for Disease Control and Prevention data collectors.
